# Angioléiomyome calcifié plantaire

**DOI:** 10.11604/pamj.2013.14.77.2477

**Published:** 2013-02-26

**Authors:** Sanaa Lemtibbet, Badredine Hassam

**Affiliations:** 1 Université Med V, Service de dermatologie, CHU Ibn Sina, Rabat, Maroc

**Keywords:** Nodule plantaire, angiolipome, Angioléiomyome, calcification, plantar nodule, angiolipoma, Angioleiomyoma, calcification

## Image en médicine

Nous rapportons le cas d'un patient de 66 ans qui consultait pour un nodule plantaire asymptomatique, augmentant progressivement de volume depuis 6 ans. L'examen retrouvait une tuméfaction du bord latéral du pied droit, de 2 cm de diamètre, de consistance dure, sensible, bien limitée, adhérente au plan superficiel et sans modification de la peau en regard. L’échographie des parties molles du pied droit objectivait un magma de calcifications groupées, mesurant 11x 6 mm, sans rapport avec les structures de voisinage notamment l'os et les tendons, de siège superficiel. Une exérèse complète de la masse était effectuée. En per-opératoire l'examen macroscopique montrait un nodule mesurant 1x1x0,8 cm, bien limité, induré, de couleur blanc jaunâtre avec à la coupe de nombreux foyers de calcifications et une capsule fine en périphérie. L'examen anatomopathologique de la pièce opératoire confirmait le diagnostic d'angioléiomyome calcifié en révélant un nodule bordé par une fine capsule fibreuse, constitué d'une prolifération vasculaire essentiellement de type capillaire, de taille variable baignent dans une prolifération musculaire lisse composée de faisceaux entrecroisés de cellules fusiformes régulières dépourvus d'atypie cytonucléaires ou de mitoses. Ces cellules se disposaient en manchons épais périvasculaires et étaient ailleurs dissociées par des zones fibreuses et par endroit calcifiées. Aucune récidive n'a été objectivée avec un recul de 3 ans. Hypothèse diagnostic: angiolipome, liposarcome; Diagnostic retenu: Angioléiomyome calcifié.

**Figure 1:**
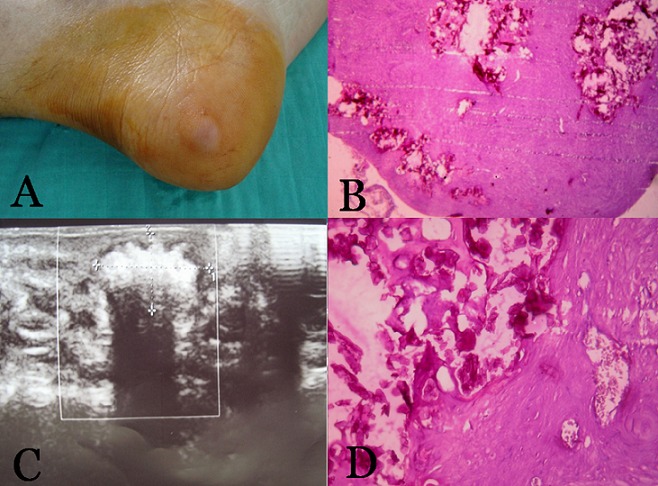
A:nodule plantaire, B:Prolifération calcifiée faite d'image en manchons périvasculaires C: aspect échographique montrant un magma de calcifications D: Prolifération fusocellulaire avec foyers de calcifications

